# Variation of the Linkage of Root Function with Root Branch Order

**DOI:** 10.1371/journal.pone.0057153

**Published:** 2013-02-25

**Authors:** Yingqian Long, Deliang Kong, Zhengxia Chen, Hui Zeng

**Affiliations:** 1 The Key Laboratory of Science and Technology of Urban Environment, Peking University Shenzhen Graduate School, Shenzhen, People’s Republic of China; 2 School of Life Sciences, Henan University, Kaifeng, People’s Republic of China; 3 Department of Ecology, College of Urban and Environmental Sciences and the Key Laboratory for Earth Surface Processes of the Ministry of Education, Peking University, Beijing, People’s Republic of China; DOE Pacific Northwest National Laboratory, United States of America

## Abstract

Mounting evidence has shown strong linkage of root function with root branch order. However, it is not known whether this linkage is consistent in different species. Here, root anatomic traits of the first five branch order were examined in five species differing in plant phylogeny and growth form in tropical and subtropical forests of south China. In *Paramichelia baillonii,* one tree species in Magnoliaceae, the intact cortex as well as mycorrhizal colonization existed even in the fifth-order root suggesting the preservation of absorption function in the higher-order roots. In contrast, dramatic decreases of cortex thickness and mycorrhizal colonization were observed from lower- to higher-order roots in three other tree species, *Cunninghamia lanceolata, Acacia auriculiformis* and *Gordonia axillaries*, which indicate the loss of absorption function. In a fern, *Dicranopteris dichotoma,* there were several cortex layers with prominently thickened cell wall and no mycorrhizal colonization in the third- and fourth-order roots, also demonstrating the loss of absorptive function in higher-order roots. Cluster analysis using these anatomic traits showed a different classification of root branch order in *P. baillonii* from other four species. As for the conduit diameter-density relationship in higher-order roots, the mechanism underpinning this relationship in *P. baillonii* was different from that in other species. In lower-order roots, different patterns of coefficient of variance for conduit diameter and density provided further evidence for the two types of linkage of root function with root branch order. These linkages corresponding to two types of ephemeral root modules have important implication in the prediction of terrestrial carbon cycling, although we caution that this study was pseudo-replicated. Future studies by sampling more species can test the generality of these two types of linkage.

## Introduction

Plant roots are a complex branch system with great heterogeneity in structure and function [1], [2], [3]. One of the difficulties in root study is how to deal with such heterogeneity and evaluate its influence on ecosystem functions. Many studies have used root branch order, the position of individual root in a root branch, to explore root functional heterogeneity since the widely reported close linkage between them [1], [4], [5], [6]. For example, in tree species, the primarily developed lower-order roots with intact cortex and frequent mycorrhizal colonization are responsible for water and nutrient uptake whereas the higher-order roots with less or no cortex and mycorrhizal colonization are mainly used for water transport [1], [2], [7].

Although a relationship between root function and root branch order has been found in many species, little attention has been given to whether and how this relationship varies among species. Uncovering different patterns of the linkage of root function with root branch can be ecologically and evolutionarily important because the formation of root branch structure is usually a result of environmental and evolutionary factors [2], [3]. It has been shown that the higher species diversity in tropical and subtropical forests compared to temperate forests can harbor larger root trait variation [6]. The great root trait variation in these ecosystems has the potential to result in a different linkage of root function with root branch order. For example, in an early study, Baylis noted that many tree species in Magnoliales (distributed mainly in tropics and subtropics) had coarse, little-branched and hairless terminal roots and named it ‘magnolioid roots’ [8], [9]. Species with this type of roots depend primarily on mycorrhizal fungi for nutrient uptake [8],[10]. It is possible that the strong dependence on mycorrhizal fungi could entail the existence of fungi in higher-order roots by delaying the appearance of continuous cork layer (CCL). This may result in the existence of cortex and mycorrhizal colonization in higher-order roots, which eventually leads to a different linkage of root function with root branch order. However, no study to date has been conducted in this regard.

Most studies on the relationship between root functions and root branch order concentrate on changes of cortex and associated mycorrhizal colonization, the critical index of absorption function. However, it remains unclear about whether traits related to the transport function, e.g. diameter and density of conduits in xylem, show different patterns along root branch order. It is widely acknowledged that these hydraulic traits are closely related to plant ecological strategies. For example, plants with larger and more conduits can grow faster and higher [11], [12], [13] but are habituated to wetter conditions because they will be confronted with greater risk of cavitation in dry environments [14], [15]. Therefore, uncovering different patterns of the hydraulic traits will provide us fresh insights into our understanding of plant adaptation to different environments.

In this study, five species were sampled in tropical and subtropical forests in South China differing in taxonomic rank (a pteridophyte, a gymnosperm and a primitive angiosperm in Magnoliaceae and two other angiosperm species) and life form (herb and trees). We examined a range of anatomical structures responsible for the absorptive and transport function in the first five order roots including cortex, stele, conduits in xylem and mycorrhizal colonization. As for the conduits, the diameter-density relationship was examined in the lower- and higher-order roots, respectively. This is because the relief of mechanical strength in lower-order relative to higher-order roots ([14], [16], [17]) can lead to different diameter-density relationships in the two root segments [14], [18]. Here, by comparing root anatomical traits and conduit diameter-density relationships among the five species, we aim to test the hypothesis that there are different linkages of root function with root branch order.

## Materials and Methods

### Study Sites and Species

The study sites were located in three tropical and subtropical forests in south China with a similar subtropical monsoon climate. Site 1 was located in Heshan Hilly Land Interdisciplinary Experimental Station (22°41′N, 112°54′E), Chinese Academy of Sciences in Guangdong province. Mean annual temperatures in this site is 21.7°C [19] and long term mean annual precipitation is 1760 mm. The wet season occurs from April to September accounting for 87.5% of precipitation and the dry season begins from October to March accounting for 12.5% of the precipitation [20]. The soil type is latosolic red soil.

Site 2 was in Wutongshan National Forest Park (22°27′–22°52′N, 113°37′–114°37′E) in Shenzhen, Guangdong province. Mean annual precipitation is 1948 mm with 75.3% falling from May to September and 24.7% falling from October to April [21]. The mean annual temperature is 22.4°C. The soil type is latosolic red soil.

Site 3 was in Jianfengling Nature Reserve (18°23′–18°50′N, 108°36′–109°05′E) in Hainan province. Mean annual precipitation is 2651 mm, and 87.7% of which occurs from May to October and 12.3% occurs from November to April [22]. The mean annual temperature is 20°C and soil type is laterite soil.

Five species with diverse plant phylogeny and life form were sampled in these sites (Table 1). Three species were in site 1 including one perennial fern, *Dicranopteris dichotoma,* one conifer, *Cunninghamia lanceolata* and one evergreen angiosperm, *Acacia auriculiformis*. A primitive angiosperm in Magnoliaceae, *Paramichelia baillonii*, was collected in site 2. Another angiosperm, *Gordonia axillaris* growing in rock crevices (personal observation) with great adaptation to infertile soil, was collected in site 3. All necessary permits in this study have been obtained from South China Botany Garden, Chinese Academy of Sciences (site 1), Xianhu Botanic Park (site 2) and Jianfengling Nature Reserve Management Bureau (site 3), respectively.

**Table 1:** Taxonomic rank, life form and habitat of five species and precipitation properties of study sites.

Species	LifeForm	Habitat	Site	Mean annualprecipitation	Rainy and dry season
PteridophytesGleicheniaceae					
* Dicranopteris dichotoma* (Didi)*	Herb	Slopes or openhillsides	Site 1	1760 mm	Rainy season^1^: Apr. to Sep.; accounting for87.5% of mean annual precipitationDry season: Oct. to Mar; accounting for12.5% of mean annual precipitation
GymmospermTaxodiaceae					
* Cunninghamia lanceolata* (Cula)	Tree	Productive, welldrained and acid soil	Site 1	1760 mm	Rainy season^1^: Apr. to Sep.; accounting for87.5% of mean annual precipitationDry season: Oct. to Mar; accounting for12.5% of mean annual precipitation
AngiospermMagnoliaceae					
* Paramichelia baillonii* (Paba)	Tree	Mountainous rainforest	Site 2	1948 mm	Rainy season^2^: May to Sep.; accounting for75.3% of mean annual precipitationDry season: Oct. to Apr.; accounting for24.7% of mean annual precipitation
Leguminosae					
* Acacia auriculiformis* (Acau)	Tree	Hygrophilous, alsoadapt to arid soil	Site 1	1760 mm	Rainy season^1^: Apr. to Sep.; accounting for87.5% of mean annual precipitationDry season: Oct. to Mar; accounting for12.5% of mean annual precipitation
Theaceae					
* Gordonia axillaris* (Goax)	Tree	Adaptable, even inharsh environment	Site 3	2651 mm	Rainy season^3^: May to Oct.; accounting for87.7% of mean annual precipitationDry season: Nov. to Apr.; accounting for12.3% of mean annual precipitation

* Letters in brackets represent the abbreviation of each species.

1 Shen *et al.*, 2000.

2 Farm *et al.*, 2002.

3 Zhou *et al.*, 2009.

### Root Sampling and Dissection

Root samples were collected in the mid growing season of 2011. Three individual trees for each species were randomly chosen. At the base of each tree trunk, one to three soil blocks (20×20×10 cm) were removed by shovels and small knives. Root segments containing at least five branch orders were collected after removal of organic matter, soil particles and dead root fragments [23]. Each sample was carefully cleaned with deionized water and was put immediately in Formalin-Aceto-Alcohol (FAA) solution (90 ml 100% ethanol, 10 ml 100% glacial acetic acid) [1].

### Anatomical Measurements

More than 10 root segments for each species were dissected by branch order. The most distal roots with no branches were defined as the first order and the roots in which two first order roots met was the second order [1]. The rest branch orders were determined in the same manner. We sampled 20, 15, 10, 10 and 10 root segments for the first to fifth order, respectively. For hard roots especially in high orders, they were softened in boiling water for one or two minutes according to its stiffness. The water-softened roots were then put in softener solution (10 ml glycerin, 10 ml aquafortis, 80 ml distilled water) for 24 hours [24]. All the root segments were dehydrated in an ethanol solution series and purified in 100% xylene before being embedded in paraffin [25]. Cross-sections of 8 µm thick were cut by a microtome (Rotary Microtome KD-2258, Zhejiang province, China). For the first-order roots, three sections near root base were selected and for higher-order roots longer than 1 cm, three sections between 1 cm from the branching point to root base were chosen [1]. After de-paraffin, they were stained with safranine and fast green. All samples were photographed under 40× to 80× magnification using a light microscope (Carl Zeiss Axioscop 20, Jena, Germany).

For each root transverse slice, a range of root anatomic traits including root diameter, stele diameter, cortex thickness, conduit lumen diameter, conduit number and conduit wall thickness were measured using Image J software (NIH Image, Bethesda, MD, USA). In addition, presence rate for mycorrhizal colonization (MC), secondary xylem (SX) and continuous cork layer (CCL) were calculated as the number of root segments bearing these structures divided by total number of roots examined for each branch order. All roots of the five species were colonized by arbuscular mycorrhizal fungi (AM) and the appearance of coils or arbuscules was regarded as infection by AM fungi [1].

Conduit diameter was calculated as the hydraulic weighted conduit diameter (*D*_h_) by the formula below [26], [27]:
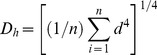
(1)where *d* was the conduit lumen diameter and *n* was the conduit number. As the majority of conduits were noncircular in shape, the conduit lumen diameter (*d*) was calculated by the average of minimal and maximal axes [27]. Conduit density was expressed as number of conduits per unit stele cross-section area in lower-order roots and in higher-order roots it was calculated based on secondary xylem transverse area. Conduit thickness-to-span ratio, a parameter reflecting the potential of conduits to resist implosion and cavitation [28], was calculated as the ratio of conduit wall thickness to mean conduit lumen diameter. Abbreviations can be found in Table S1.

### Statistical Analysis

The first five branch orders were examined in four tree species except in *D. dichotoma* with only four branch orders. Root anatomical traits, e.g., diameter of root, stele and conduit, cortex thickness, stele to root diameter ratio and conduits density, were transformed logarithmically to meet the normal distribution when necessary. In each species, differences of these traits among branch orders were analyzed by one-way ANOVA and Tukey’s HSD test was used for uneven sample size [1].

The relationship between root function and root branch order was assessed by hierarchical cluster analyses with the pairwise rescaled distance or similarity. This analysis was conducted in each species as well as all the five species together to explore variations of the linkage of root function with root branch order. Root traits included in these analyses were cortex thickness, stele to root diameter ratio, rate of mycorrhizal colonization, and presence rate of secondary xylem and continuous cork layer.

The relationship between conduit size and conduit density was assessed by linear regressions in lower- and higher-order roots, respectively. These two root segments were separated by the presence of secondary xylem (SX). For example, the lower-order roots referred to roots with no or poor SX and the higher-order roots were those with significant presence of SX. In order to explore variations of the conduit diameter-density relationship among species, slopes of these regressions were compared by standardized major axis (SMA) using the SMART software [29]. As the conduit diameter-density relationship was rather weak in lower-order roots, variations of this relationship were evaluated by comparing coefficient of variance (CV) for these two traits in each species. The comparison of CV was conducted by comparing the component of CV including variance and mean value. Before the comparison, data were transformed by dividing the maximum value of lower-order roots in each species because the dimensions of conduit diameter and density were different (Table S2). Variances of these two traits were compared by testing homogeneity of variances using Levene statistic. Comparison of the mean values was conducted with independent t-test. All analyses were conducted in SPSS (version 13.0; SPSS Inc. Chicago, USA). The significant level was set at 0.05.

## Results

### Root Anatomic Traits

**Figure 1:**
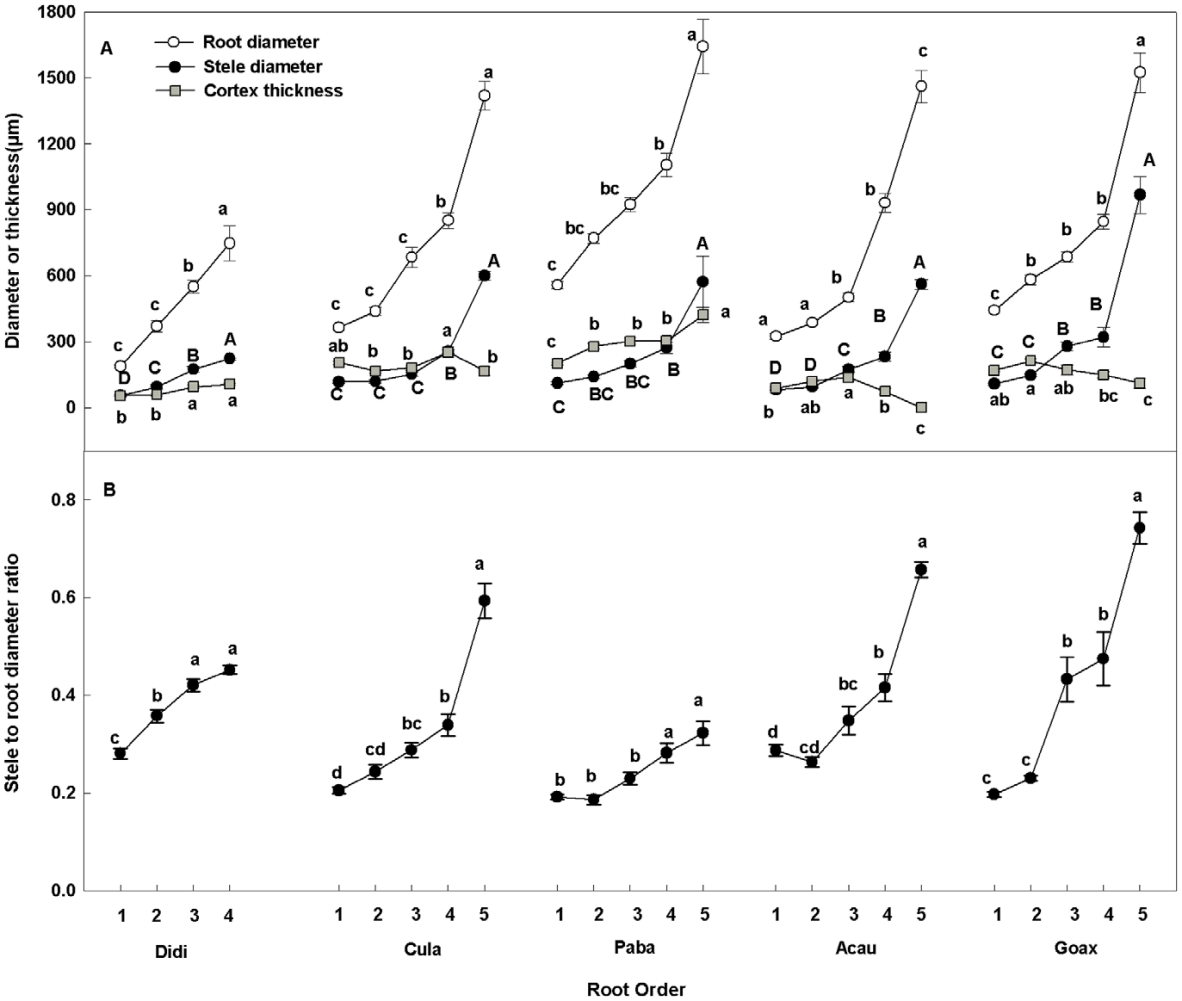
Root anatomical traits along five orders for five species. Root diameter, stele diameter and cortex thickness (A), and stele to root diameter ratio (B) for the first five root orders in five species. (A) Root and stele diameter are indicated by open circle and closed circle respectively, and cortex thickness are shown by grey square. Error bars represent one standard error of the mean. Significant difference (*P*<0.05) for a trait among root orders within individual species is indicated by different lower or upper case letters. See Table 1 for species abbreviations.

Root diameter, stele diameter (Fig. 1A) and stele to root diameter ratio (Fig. 1B) increased with branch order in each of the five species. Cortex thickness increased continuously with branch order in *D. dichotoma* and *P. baillonii* whereas it decreased from a certain order in *C. lanceolata*, *A. auriculiformi* and *G. axillaris* (Fig. 1A). In *D. dichotoma*, there was a remarkable wall-thickened cortex in the third and fourth orders (Fig. S1).

**Figure 2:**
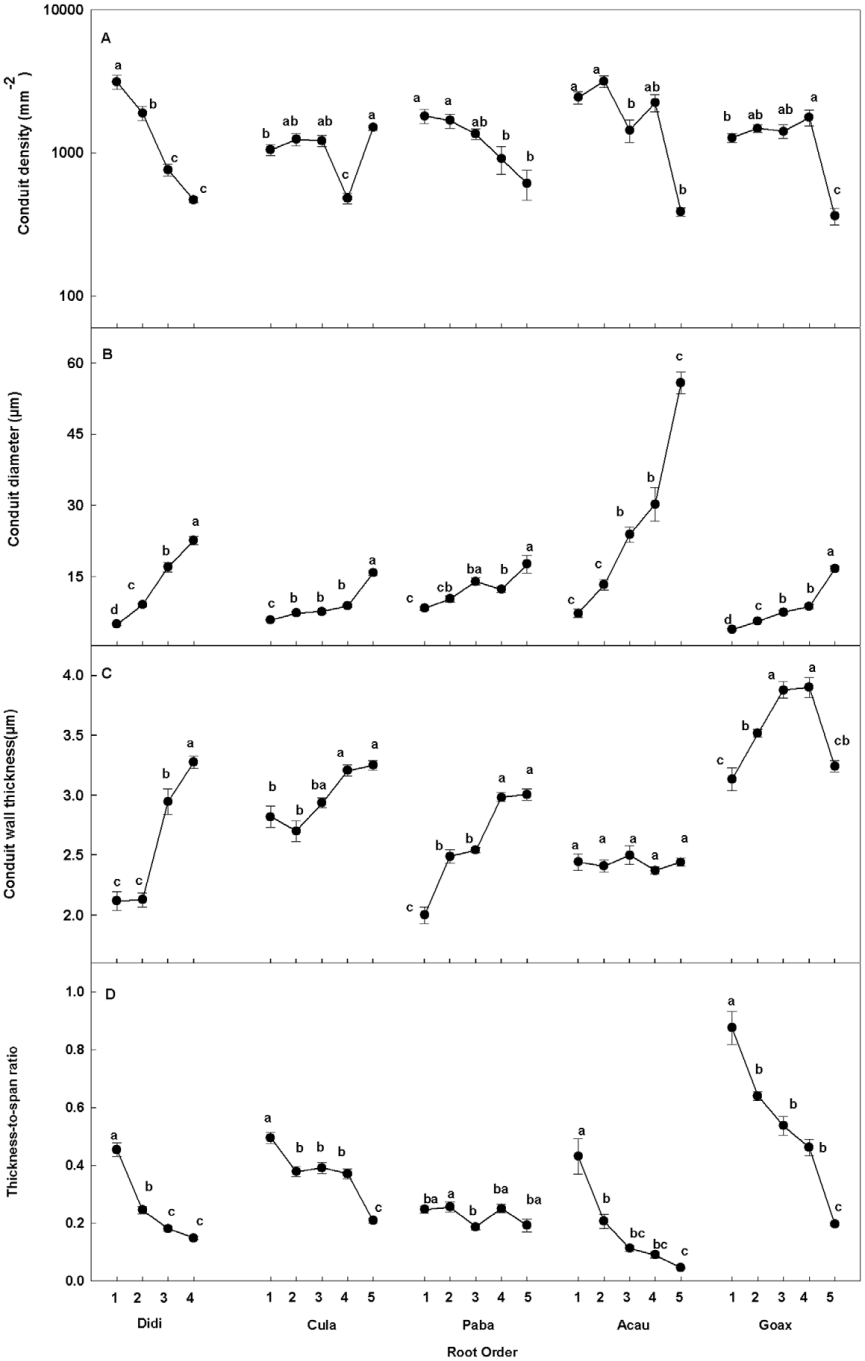
Conduit traits along five orders for five species. Conduit density (A), conduit diameter (B), conduit wall thickness (C) and conduit thickness to span ratio (D) among the first five orders of five species. Error bars represent one standard error of the mean. Lowercase letters that differ among root branches within a species indicate a significant difference (*P*<0.05). Abbreviations for species are given in Table 1.

Conduit density was lower in higher-order roots in species except for *C. lanceolata* exhibiting 3-fold increase from the fourth- to the fifth-order roots. In *A. auriculiformi,* conduit diameter had 7.7-fold increase from the first- to fifth-order root (Fig. 2B). Conduit wall thickness increased with root branch order in *D. dichotoma*, *C. lanceolata*, and *P. baillonii* whereas decreased from the fourth to fifth orders in *G. axillaris*. In contrast, there was a similar conduit wall thickness among root branch order of *A. auriculiformi* (*P*>0.05, Fig. 2C). The conduit thickness-to-span ratio decreased with root branch order in all the five species except *P. baillonii* which had little variation among the five branch orders (Fig. 2D). In the first-order roots, *P. baillonii* had the largest conduit diameter (8.0 µm), the thinnest conduit wall (2.0 µm) and the largest thickness-to-span ratio whereas *G. axillaris* had the smallest conduit diameter (4.0 µm), the thickest conduit wall (3.2 µm) and the smallest conduit thickness-to-span ratio (Fig. 2B,C,D).

Mycorrhizal colonization (MC) decreased with root branch order (Table 2). No or little MC occurred from the fourth- to fifth-order roots except in *P. baillonii* with a MC rate of 0.4 in the fifth-order roots. The presence rate of CCL was similar to that of SX in species except for *P. baillonii* demonstrating a rather poor presence rate in fifth-order root (Table 2). Secondary xylem appeared in higher-order roots in species except for *D. dichotoma* devoid of secondary development.

**Table 2:** The presence rate of mycorrhizal colonization (MC), secondary xylem (SX) and continuous cork layer (CCL) by root orders within each species.

	*D.dichotoma*			*C. lanceolata*			*P. baillonii*			*A. auriculiformis*			*G. axillaris*		
	MC	SX	CCL	MC	SX	CCL	MC	SX	CCL	MC	SX	CCL	MC	SX	CCL
Order1	0.80	−	−	0.60	−	−	0.90	−	−	0.61	−	−	0.88	−	−
Order2	0.53	−	−	0.33	−	−	0.87	−	−	0.43	0.07	−	0.67	−	−
Order3	0.09	−	−	0.25	−	−	0.80	0.20	−	0.25	0.79	0.25	0.27	0.41	0.18
Order4	−	−	−	−	0.57	−	0.55	0.60	−	0.05	1	1	−	0.88	0.75
Order5	−	−	−	−	1	0.70	0.40	0.90	0.15	−	1	1	−	1	1

### Cluster of Root Anatomic Traits

**Figure 3:**
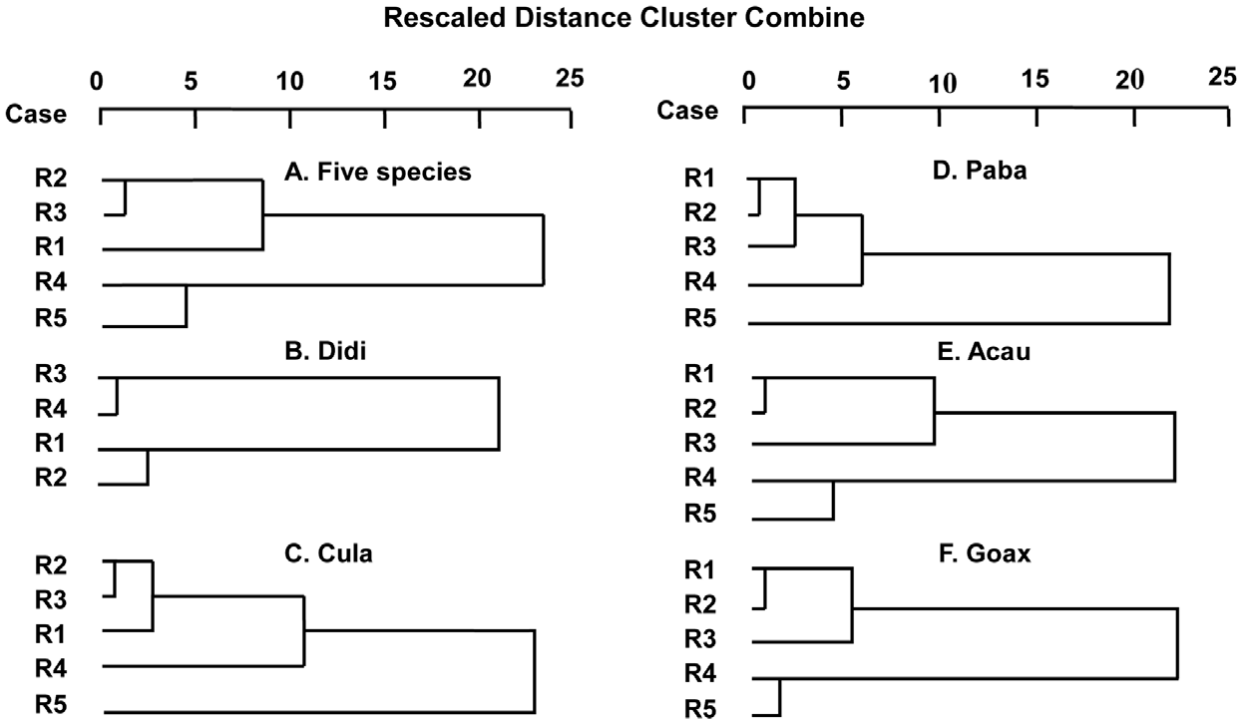
Results of clustering analysis of root branches. The Results of hierarchical clustering analysis of root orders for total species (A) and each of the five species (B-F). Clusters of root orders are based on the rescaled distance for the close pairwise distance indicating these orders are similar to each other. R1to R5 represents the first to the fifth root orders. Abbreviations for species are given in Table 1.

Average across the five species, the first five orders were divided into the lower- (the first three orders) and higher-order (the fourth and fifth orders) root segments (Fig. 3A). Individually, the first two or three orders were clustered into the lower-order root segments in four of the five species (Fig. 3B,C,E,F). In contrast, the lower-order root segment included the first four orders in *P. baillonii* (Fig. 3D).

### Relationships between Conduit Diameter and Density

**Figure 4:**
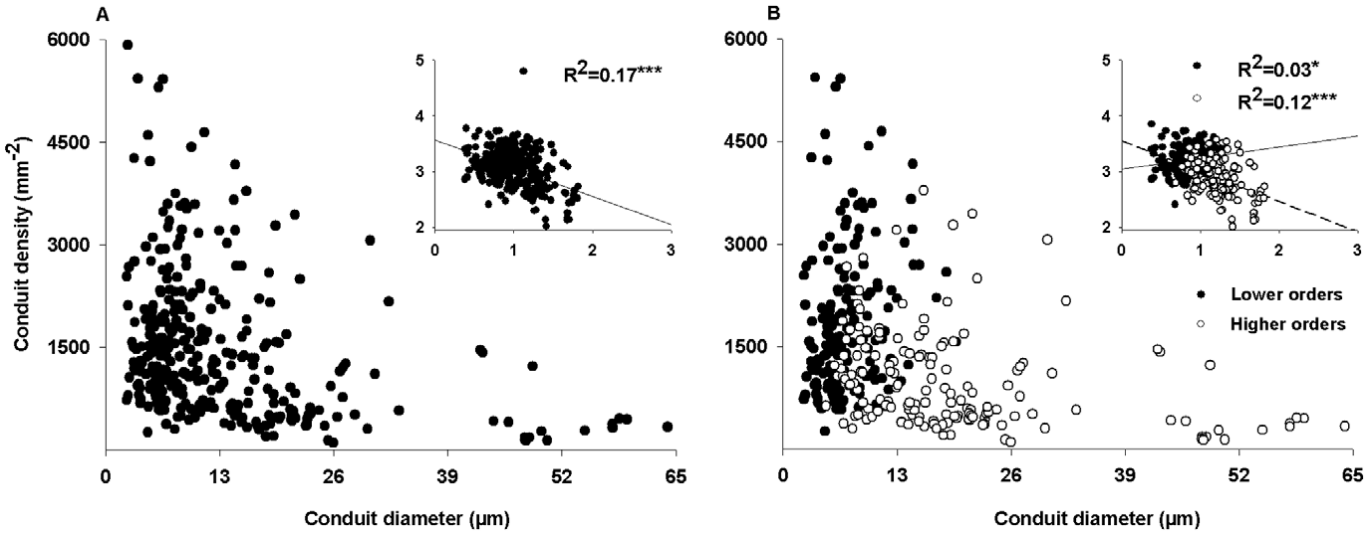
Relationships between conduits density and diameter. The relationships between conduits density and hydraulic weighted conduit diameter for all species (A), lower orders (closed circles) and higher orders (open circles) (B). See Figure 5 for the lower and higher orders’ classification of each species. Linear regressions with log-transformed data are as detailed in insets with solid line for all species (A) and the lower orders (B), and with dash line for the higher orders in (B). *, **and *** are the significant level at 0.05, 0.01 and 0.001, respectively.

**Figure 5:**
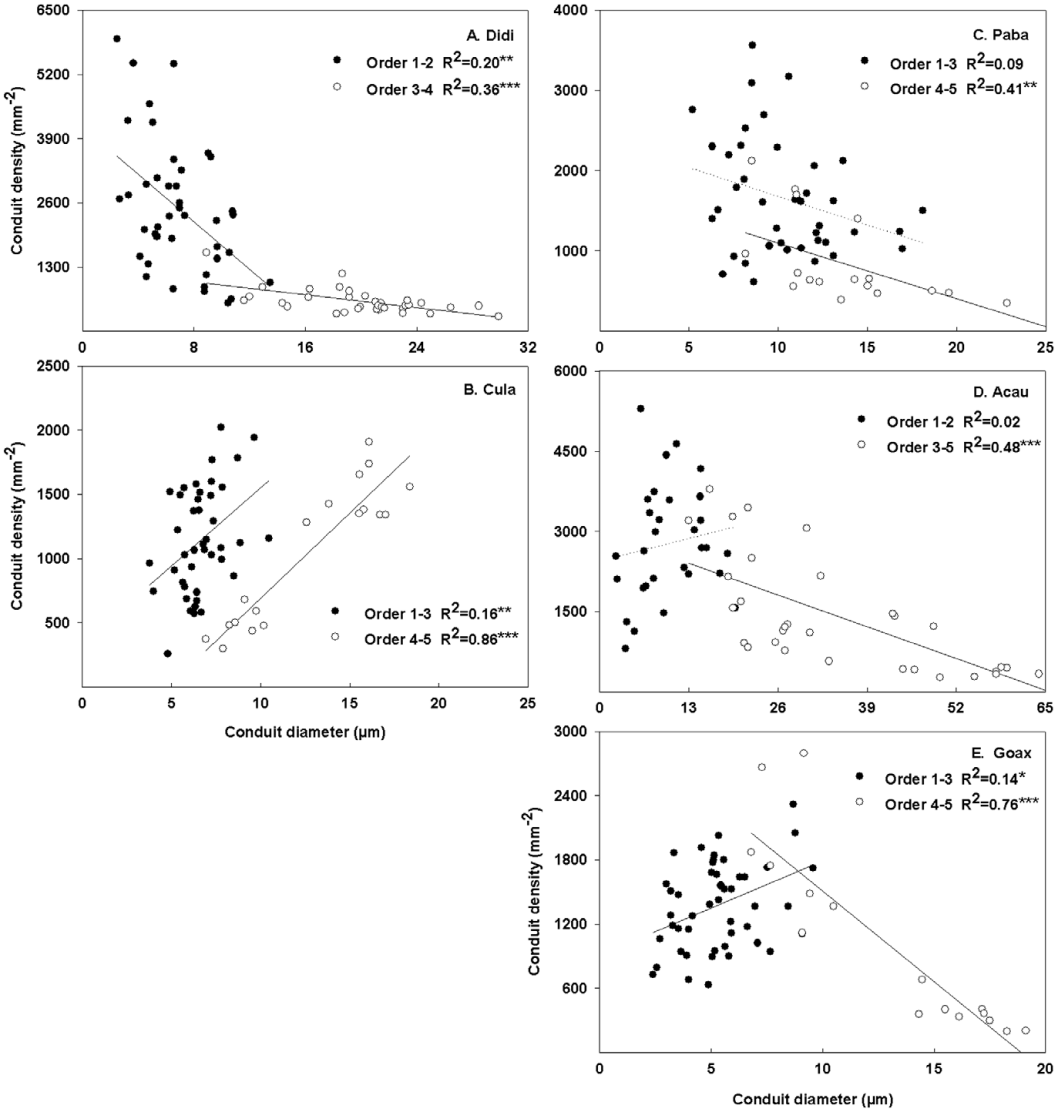
Relationships between conduits density and diameter for each species. The relationships between conduits density and hydraulic weighted conduit diameter for lower and higher orders in each of five species. The lower (closed circles) and higher orders (open circles) are classified by the presence of secondary xylem. Significant regressions are indicated as solid lines and dotted line are noted as non-significant regressions. *, **and *** represent the significant level at 0.05, 0.01 and 0.001, respectively. See Table 1 for species abbreviations.

For all the five species, conduit density was negatively correlated with conduit lumen diameter (Fig. 4A). When considered separately, the conduit diameter-density relationship was stronger in lower- than higher-order roots (Fig. 4B). The negative relationships in higher-order roots are stronger in four (Fig. 5A, C, D, E) of the five species except *C. lanceolata* with a positive relationship (Table S2; Fig. 5B). The slopes of the four negative relationships followed in a sequence as *G. axillaris* <*P. baillonii*<*A. auriculiformi* = *D. dichotoma* (Table S2,S3).

The conduit diameter-density relationships were much lower in lower-order than higher-order roots (Fig. 5). Averaged across the five species, CV of conduit diameter (44.7%) was smaller than that of conduit density (62.0%, Table 3). The higher CV of conduit density than conduit diameter occurred in *D. dichotoma*, *C. lanceolata*, *P. baillonii* but not in *A. auriculiformi* and *G. axillaris* (Table 3, S4). In *D. dichotoma* and *P. baillonii*, the higher CV of conduit density than conduit diameter was contributed by their lower mean density whereas the higher CV of conduit density in *C. lanceolata* was a result of the higher variance of conduit density (Table S4). The patterns of CV were more prominent when only first-order roots were considered (Table S5).

**Table 3:** Coefficient of variance (CV) for conduit diameter and density in lower orders.

	CV-diameter	CV- density	Ratio of CV^b^
Lower orders for total five species^a^	44.69%	61.97%	1.39
Lower orders for Didi	26.90%	59.50%	2.21
Lower orders for Cula	20.45%	35.93%	1.76
Lower orders for Paba	28.70%	43.58%	1.52
Lower orders for Acau	44.99%	38.76%	0.86
Lower orders for Goax	33.06%	29.60%	0.90

a Lower orders refer to root orders with no secondary xylem. See Table 1 for species abbreviation.

b Ratio of CV is calculated by CV of density divided by CV of diameter.

## Discussion

### Variation of the Linkage Indicated by Root Cortex

Mounting evidence has revealed a tight linkage between root function and root branch order [1], [3], [4], [5], [30]. This can be demonstrated for the water transport function in the five species encompassing diverse plant phylogeny and life form (Table 1). With increasing root branch order, there were larger steles, wider conduits and higher stele to root diameter ratio (Fig. 1, 2B). Variations of these traits indicate an increase of water transport capacity along root branch order because the potential hydraulic conductivity is related to the fourth power of conduit diameter [27], [31]. The enhancement of water transport function was accompanied the loss of absorptive function in four species. This was indicated by the dramatic decrease of cortex thickness from a certain branch order of *C. lanceolata, A. auriculiformi* and *G. axillaries* (Fig. 1A) as the significant role of cortex in resource absorption and mycorrhizal colonization [7], [32], [33], [34]. In *D. dichotoma*, it may also have no absorptive function in the higher-order roots despite a consistent increase of cortex thickness with branch order. This is because the wall-thickened cortex cell layers (Fig. S1), similar to the schlerenchyma fiber in fern stipes [35], [36], can function as a protective tissue to prevent water loss and MC in these roots [37], [38]. The functional transition from lower- to higher-order roots in the four species represents the common relationship between root function and root branch order [1], [2].

Different from the four species above, there was a continuous increase of cortex thickness associated with considerable MC in higher-order roots of *P. baillonii* (Table 2; Fig. 1A). This indicates clearly the existence of absorptive function in these roots. Obviously, the linkage of root function with branch order in *P. baillonii* is different from that in four other species, which supports our hypothesis. This can also be supported by the cluster analysis which showed a different pattern of branch order classification in *P. baillonii* from that in other species (Fig. 3). The different patterns of linkage of root function with root branch order may result from the different relationships between two secondary tissues, SX and CCL, a hydrophobic and protective layer (Table 2) [1], [7], [39]. Generally, the loss of absorptive function in higher-order roots can be attributed to the extrusion of cortex by these secondary tissues, which may be the case for the above four species. However, there was a prominent lag for the appearance of CCL behind SX in *P. baillonii* (Table 2; Fig. S1). This can in turn result in the increase of cortex thickness accompanied with the presence of MC in higher-order roots.

A possible explanation for the asynchrony of CCL and SX in *P. baillonii* lies in the multiple functions undertaken by the cortex. As for root cortex, it can function as carbon storage as well as the usually acknowledged absorptive function. This can be the case for *P. baillonii*, which has the coarse, little-branched and hairless ‘magnolioid roots’ [8], [10], [40]. In this type of root, nutrient uptake depends primarily on mycorrhizal fungi [8], [9]. This can be supported by the high MC rate (Table 2) and extent as indicated by the thicker cortex that can host more mycorrhizal fungi [1], [36] in *P. baillonii*. The strong symbiosis with mycorrhizal fungi will entail more carbon supply because fungi construction and maintenance of fungi activity are energy-expensive [41], [42], [43]. Thus, carbon storage in root cortex as demonstrated in many other studies [27], [38] can be a convenient way to meet the carbon demand from mycorrhizal fungi. This speculation is also supported by the equal carbon (C) concentration among the first five order roots (unpublished data). Generally, root C concentration increases in higher-order roots because of the larger C requirements of mechanical strength and water transport function in these roots [27], [44]. However, this root C pattern can be altered in ‘magnolioid roots’ when parenchyma cells in the thick cortex serve as carbon storage (for example, starch) [38], [45] and the proportion of cortex in root cross section area was higher in lower- than higher-order roots (Fig. 1B). This will make C concentration in lower-order root of *P. baillonii* high enough to be equal as that in higher-order root.

### Variation of the Linkage Indicated by Conduit Traits

Besides the cortex, the pattern of conduit diameter-density relationship can also support the different linkages of root function with root branch order. In higher-order roots, our results showed diverse conduit diameter-density relationships in direction and strength for the five species (Table S2, S3; Fig. 5). Despite these diverse relationships, they may be underpinned by two different mechanisms. In *D. dichotoma*, *A. auriculiformis* and *G. axillaries*, the negative conduit diameter-density relationships in higher-order roots (Table S2,S3; Fig. 5) can be explained by the compromise of water transport with root function for mechanical strength besides the safety-efficiency tradeoff in water transport [14], [18], [27]. This can be indicated by the significant investment in mechanical structures such as the thickened cortex wall in *D. dichotoma* (Fig. S1A) and fiber in higher-order roots of *A. auriculiformis* (see Fig. S1D) and *G. axillaries* (see Fig. S1E). The higher investment in mechanical strength in *G. axillaries* (as indicated by the thicker fiber) than in *A. auriculiformis* (Fig. S1) can also contribute to the faster reduction of conduit density when increasing conduit diameter in the former than the latter (see slope comparison in Table S2,S3; Fig. 5). Given the limited potential of tracheids to expand as wide as vessels [46], [47] the positive conduit diameter-density relationship in *C. lanceolata* may reflect its strategy of enhancing water transport by the specialized torus-margo pit membrane with higher inter-conduit conductance [48], [49], [50].

Different from the four species above, the negative conduit diameter-density relationship in *P. baillonii* can be attributed to mechanisms other than mechanical strength because there was little fiber even in the fifth-order root (Fig. S1). As discussed previously, this relationship can also result from a tradeoff of carbon allocation between hydraulic structures in the stele and carbon storage parenchyma in the cortex [14], [18], [27]. Thus, the different mechanisms underlying the different conduit diameter-density relationships in higher-order roots provide further support the two types of linkage between root function and root branch order that were initially revealed by variation of root cortex.

Consistent with the patterns of hydraulic relationship in higher-order roots, CV of conduit diameter and density in lower-order roots of *P. baillonii* also showed a different pattern from other species (Table 3,S4). In lower-order roots of *P. baillonii*, CV of conduit diameter was lower than that of conduit density. Analysis of the two components of CV showed that the lower CV of conduit diameter was due to a higher mean value of conduit density than that of conduit density rather than a higher variance of conduit diameter than that of conduit density because the variances of conduit diameter and density were equal (). The similar variance for conduit density and diameter in *P. baillonii* (Table S4) may result from their conduits, larger in diameter and thinner in wall than other species (Fig. 2B, C). This is because if conduits with large diameter and thin wall, e.g. in *P. baillonii*, had great variation they would be confronted with high risk of conduit cavitation and imploding in dry seasons. However, the other four species, all characterized by small conduit diameter and thick walls (Fig. 2B, C, D) may depend mainly on conduit density (e.g. *D. dichotoma* and *C. lanceolata*) or on both conduit diameter and density (e.g. *A. auriculiformi* and *G. axillaris*) (Table 3,S4) to handle dry seasons (Table 1). Thus, different variation patterns for conduit diameter and density in lower-order roots constitute further evidence for different patterns of the relationship between root function and root branch order.

### Different Ephemeral Root Modules

The different linkages of root function with root branch order can correspond to different types of ephemeral root modules. The root modularity, a conception proposed two decades ago but tested only recently, depends on independent function and life history (as indicated by different lifespan) of one root segment from another root segment [3]. Although root lifespan was not measured, differences of anatomical traits between lower- and higher-order root segments as presented above can reflect ephemeral property of the lower-order root segment. This is because primarily developed tissues in this root segment are more palatable to herbivores or more easily to lose function in adverse environments [51], [52].

Although the ephemeral root module is not necessarily confined to the first three orders in a root branch [53], little is known about how the ephemeral root module varies in different species. Results of our study indicates two types of ephemeral root module with one including both lower- and higher-order roots (e.g. *P. baillonii*) and the other including only the lower-order roots (e.g. four other species). Classification of root branch order into different types of root module may lead to different estimation of carbon cycling rate because roots of different orders usually have different carbon content and turnover rate [42], [53]. Thus, identifying different types of root module can have important implication in estimating carbon cycling rate. Furthermore, different root modules may be a result of different evolutionary and environmental factors. Uncovering these factors can enhance our understanding of plant evolution as well as the prediction of species composition and distribution in the ongoing changing climate [8], [10], [54].

### Conclusions

By examining five species encompassing diverse plant phylogeny and growth form, we revealed for the first time two types of linkages of root function with root branch order. Classification of the two linkages was supported by the variation of cortex and conduit traits which are responsible for absorptive and transport function, respectively. The two types of linkage can correspond to different ephemeral root modules and have important implications in predicting terrestrial carbon cycling. Results of this study are illuminative in our understanding of the relationship between root function and branch order. Future studies by sampling more species can test generality of two types of linkage as well as uncovering more types of the linkage of root function with root branch order.

## Supporting Information

Figure S1 Light micrographs of higher-order root transverse sections for five species.
(JPG)

Table S1 Summary of root traits for the five species in this study.
(JPG)

Table S2 Coefficient of variance (CV) for conduit diameter and density in the first root order.
(JPG)

Table S3 Comparison of SMA slopes for relationships between conduit diameter and density in higher-order root segments among the five species.
(JPG)

Table S4 Comparison of the component of CV.
(JPG)

Table S5 Coefficient of variance (CV) for conduit diameter and density in the first order.
(JPG)
