# Supplementary figures and images for: Variation of the Linkage of Root Function with Root Branch Order
Source: PLoS One. 2013 Feb 25;8(2):e57153. doi: 10.1371/journal.pone.0057153 (PMC3581569; doi:10.1371/journal.pone.0057153)

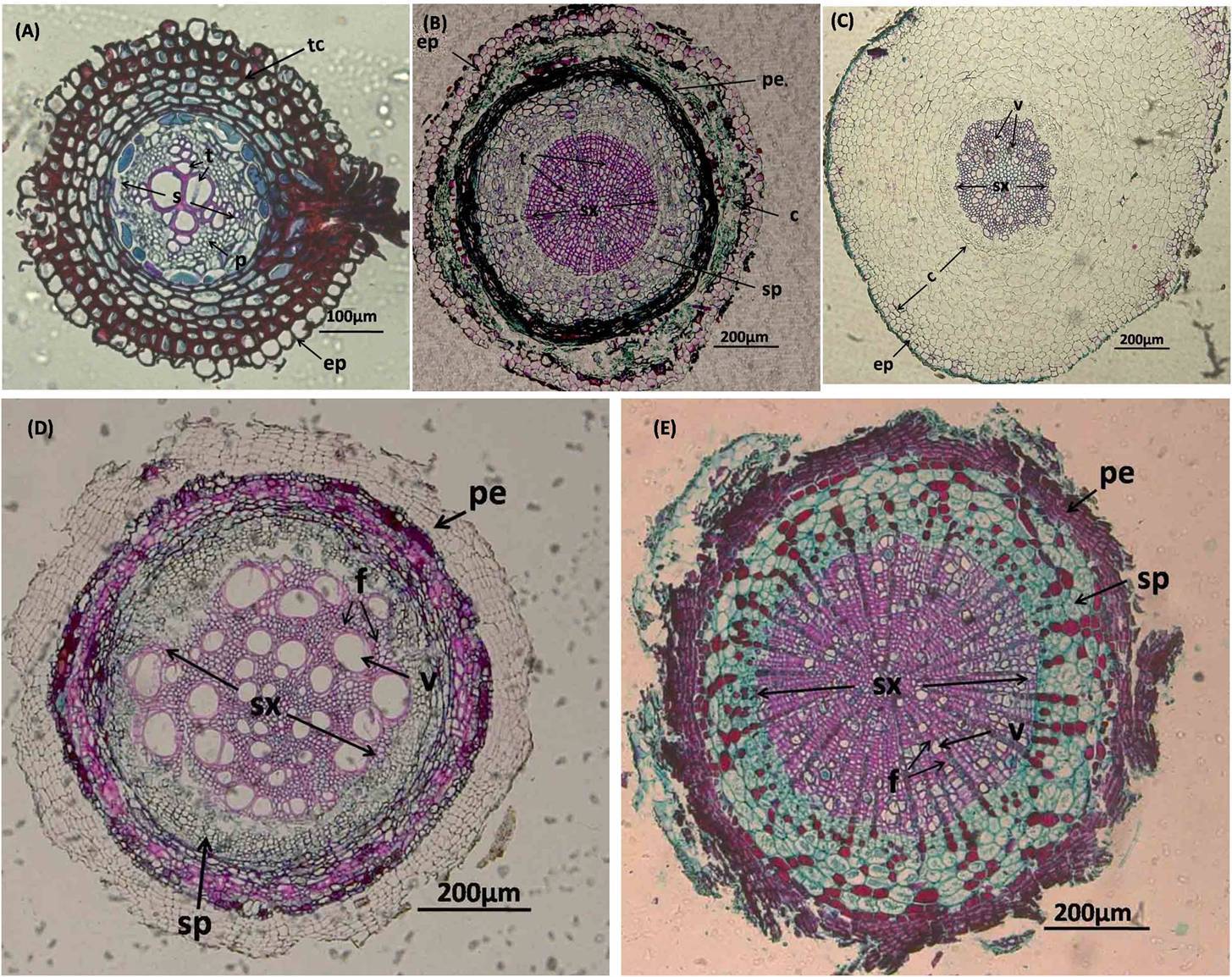

Supplement: Figure S1 — Light micrographs of higher-order root transverse sections for five species. (JPG) [file pone.0057153.s001.jpg]

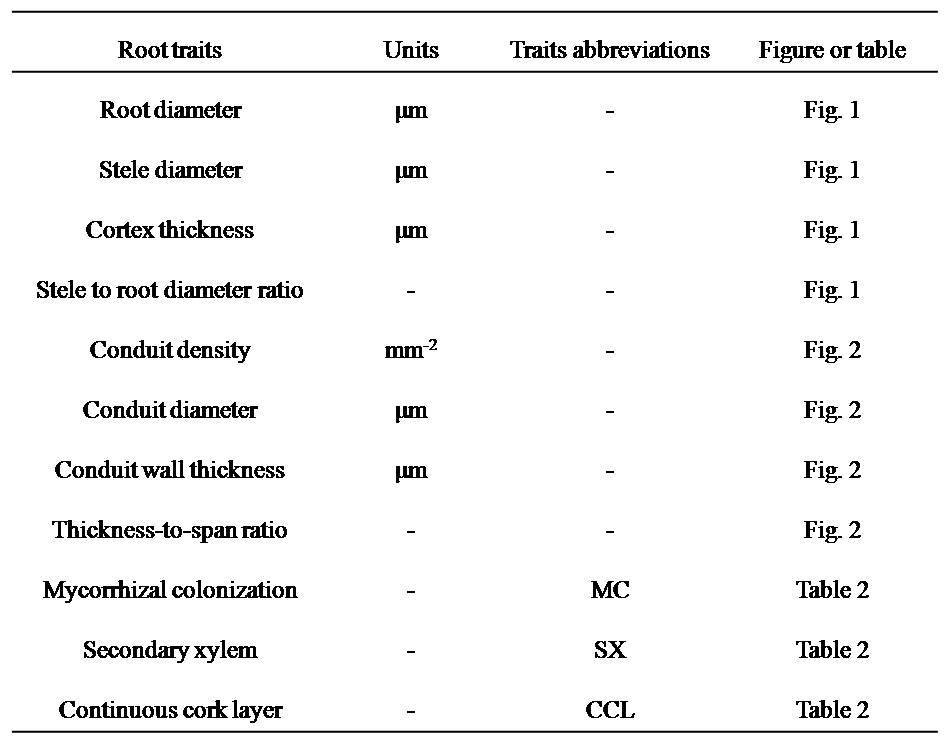

Supplement: Table S1 — Summary of root traits for the five species in this study. (JPG) [file pone.0057153.s002.jpg]

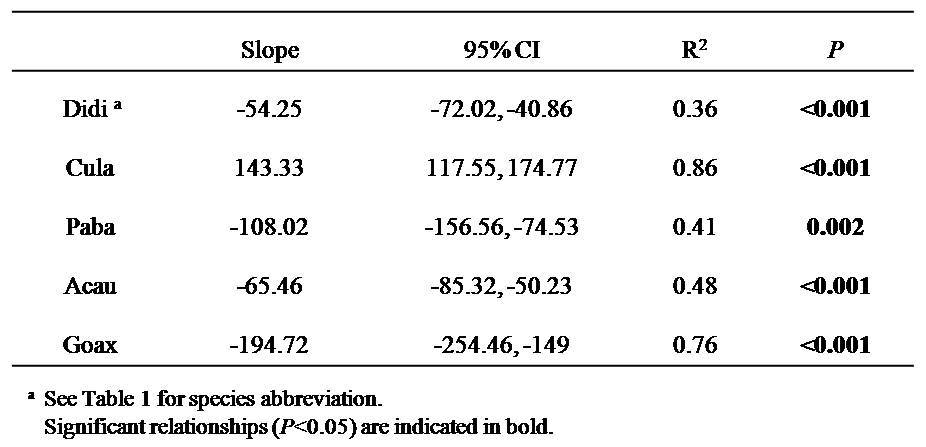

Supplement: Table S2 — Coefficient of variance (CV) for conduit diameter and density in the first root order. (JPG) [file pone.0057153.s003.jpg]

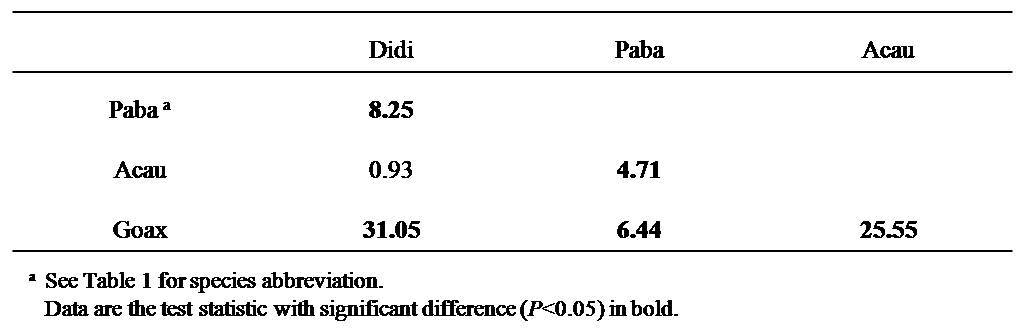

Supplement: Table S3 — Comparison of SMA slopes for relationships between conduit diameter and density in higher-order root segments among the five species. (JPG) [file pone.0057153.s004.jpg]

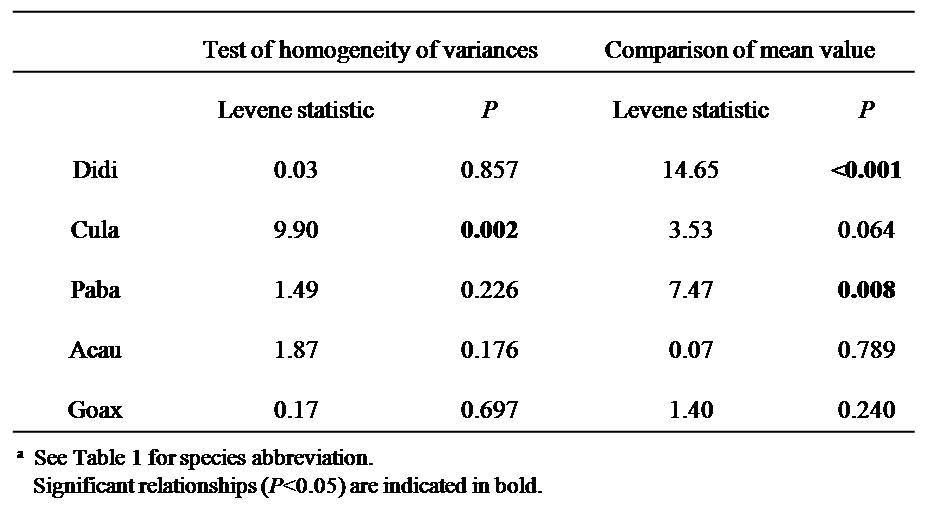

Supplement: Table S4 — Comparison of the component of CV. (JPG) [file pone.0057153.s005.jpg]

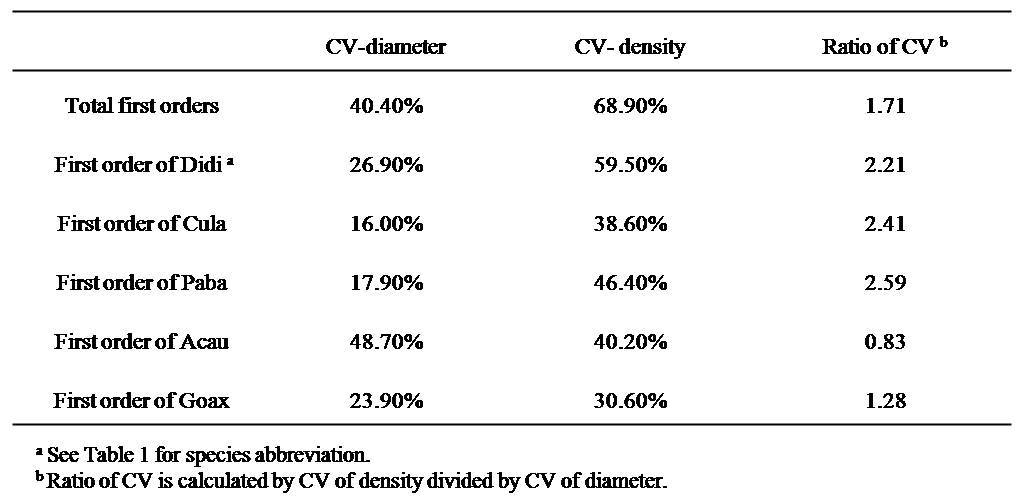

Supplement: Table S5 — Coefficient of variance (CV) for conduit diameter and density in the first order. (JPG) [file pone.0057153.s006.jpg]
